# Cost-effectiveness analysis of radiotherapy techniques for whole breast irradiation

**DOI:** 10.1371/journal.pone.0248220

**Published:** 2021-03-08

**Authors:** Yibo Xie, Beibei Guo, Rui Zhang

**Affiliations:** 1 Medical Physics Program, Department of Physics and Astronomy, Louisiana State University, Baton Rouge, Louisiana, United States of America; 2 Department of Experimental Statistics, Louisiana State University, Baton Rouge, Louisiana, United States of America; 3 Department of Radiation Oncology, Mary Bird Perkins Cancer Center, Baton Rouge, Louisiana, United States of America; URCEco Ile de France Hopital de l’Hotel Dieu, FRANCE

## Abstract

**Background:**

The current standard of care (SOC) for whole breast radiotherapy (WBRT) in the US is conventional tangential photon fields. Advanced WBRT techniques may provide similar tumor control and better normal tissue sparing, but it is controversial whether the medical benefits of an advanced technology are significant enough to justify its higher cost.

**Objective:**

To analyze the cost-effectiveness of six advanced WBRT techniques compared with SOC.

**Methods:**

We developed a Markov model to simulate health states for one cohort of women (65-year-old) with early-stage breast cancer over 15 years after WBRT. The cost effectiveness analyses of field-in-field (FIF), hybrid intensity modulated radiotherapy (IMRT), full IMRT, standard volumetric modulated arc therapy (STD-VMAT), multiple arc VMAT (MA-VMAT), non-coplanar VMAT (NC-VMAT) compared with SOC were performed with both tumor control and radiogenic side effects considered. Transition probabilities and utilities for each health state were obtained from literature. Costs incurred by payers were adopted from literature and Medicare data. Quality-adjusted life years (QALYs) and incremental cost-effectiveness ratio (ICER) were calculated. One-way sensitivity analyses and probabilistic sensitivity analyses (PSA) were performed to evaluate the impact of uncertainties on the final results.

**Results:**

FIF has the lowest ICER value of 1,511 $/QALY. The one-way analyses show that the cost-effectiveness of advanced WBRT techniques is most sensitive to the probability of developing contralateral breast cancer. PSAs show that SOC is more cost effective than almost all advanced WBRT techniques at a willingness-to-pay (WTP) threshold of 50,000 $/QALY, while FIF, hybrid IMRT and MA-VMAT are more cost-effective than SOC with a probability of 59.2%, 72.3% and 72.6% at a WTP threshold of 100,000 $/QALY, respectively.

**Conclusions:**

FIF might be the most cost-effective option for WBRT patients at a WTP threshold of 50,000 $/QALY, while hybrid IMRT and MA-VMAT might be the most cost-effective options at a WTP threshold of 100,000 $/QALY.

## Background

Breast cancer has the highest incidence rate among women in the United States besides skin cancer (www.cancer.org). Lumpectomy is commonly performed for patients with early-stage breast cancer, and whole breast radiotherapy (WBRT) after lumpectomy can improve local control and overall survival [1].

The current standard of care (SOC) for whole breast radiation therapy (WBRT) in the US and in our clinic is conventional tangential photon fields [2, 3]. Other advanced technologies had been proposed for WBRT and shown auspicious results, such as field-in-field (FIF) technique [4, 5], hybrid Intensity modulated radiation therapy (IMRT) [6–8], fixed beam IMRT [9, 10], standard volumetric modulated arc therapy (STD-VMAT) [11], multiple arc VMAT (MA-VMAT) [12]. Non-coplanar VMAT (NC-VMAT) has been shown to improve organs at risk (OAR) dosimetry for post-mastectomy breast cancer [13], but has not been investigated for WBRT. These advanced technologies are superior to SOC in improving the dose homogeneity within the target volume and reduce therapeutic dose to radiosensitive organs, but may increase low-dose cloud which could cause higher risk of radiogenic side effects [14, 15].

It has been reported that the cost of breast cancer treatments is around $16.5 billion in the United States in 2010, which is higher than any other type of cancer, and is projected to reach $20 billion by 2020 [16]. Although more advanced radiotherapy technologies may improve dosimetric outcomes under certain circumstances, their much higher cost may not justify their advantages. There have been some cost-effectiveness studies comparing partial breast irradiation and WBRT [17–19], but the comprehensive cost-effectiveness comparison among advanced WBRT techniques including costs of radiogenic side effects is still lacking.

The aim of this study was to analyze the cost-effectiveness of various WBRT techniques including conventional SOC, FIF, hybrid IMRT, IMRT, STD-VMAT, MA-VMAT and NC-VMAT. The conventional SOC was used as the reference for modality comparisons. Both tumor coverage and late side effects (cardiac toxicity and secondary cancers) after WBRT were included in the analyses.

## Methods

### Decision model

**Fig 1:**
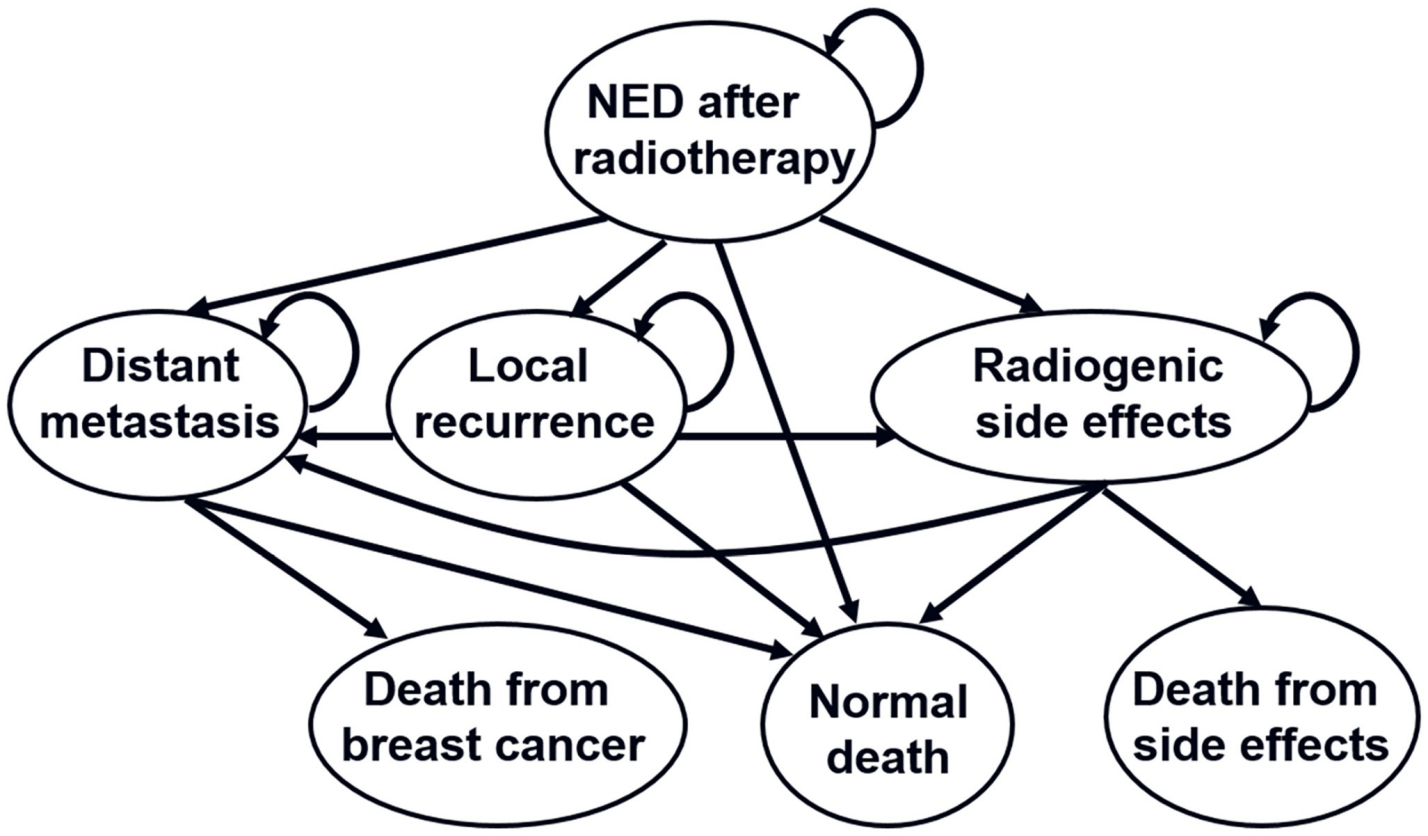
Overview of the Markov model. NED = no evidence of disease.

A Markov model (Fig 1) was designed using TreeAge Pro (Williamstown, MA) to simulate the clinical history of 65-year-old postmenopausal women with early-stage breast cancer who received lumpectomy and subsequent WBRT with a prescribed dose of 50 Gy in 25 fractions. All patients start with no evidence of disease (NED) after WBRT, and transition afterwards to one of the four states (distant metastasis, local recurrence, late radiogenic side effects, and death from other causes) in 1-year cycles. The patients could also die from breast cancer, which is mainly caused by distance metastasis, and die from radiogenic side effects.

A 15- year horizon after radiotherapy was analyzed in the model since study has shown the improvement in local control and survival due to radiotherapy during this time period [1]. The incremental cost effectiveness ratio (ICER) was expressed in terms of cost per life-year gained according to the following formula:

$$ICER=\frac{C_{1}-C_{0}}{E_{1}-E_{0}}$$

where C_0_ and E_0_ are the cost and quality adjusted life year (QALY) for SOC technique, and C_1_ and E_1_ are the cost and QALY for other WBRT techniques. Willingness-to-pay (WTP) thresholds of $50,000/ QALY and $100,000/QALY [20, 21] were used to determine whether a WBRT technique is cost-effective.

### Model data input

All transition probabilities and utilities for SOC were extracted from published literature [1, 22–26] and are shown in Table 1.

**Table 1:** Annual transition probability and utility for the 65-year-old patient cohort who received SOC WBRT.

Parameters	Years	Value (%) (range)	Reference
**Probability**			
Local Recurrence	0–5	1.46	[1]
	6–10	0.54	
	11–15	0.06	
NED to metastasis	0–5	12.25	[22]
	6–10	7.75	
	11–15	7.75	
Metastasis to Death	0–5	23.2	[23]
	6–15	14.0	
Death from other causes	65–70 (age)	1.46	[27]
	71–75 (age)	1.71	
	76–80 (age)	3.53	
Death due to lung toxicity	11–15	0.078	[25]
Death due to heart toxicity	11–15	0.53	[26]
Death due to CL breast toxicity	6–15	2.12	[1]
**Utility**			
NED	0–5	0.734	[28]
	6–10	0.716	
	11–15	0.675	
Cardiac toxicity	11–15	0.57(0.54–0.61)	[29]
CL Breast cancer	6–15	0.54(0.48–0.55)	[30]
Lung cancer	11–15	0.50 (0.39–0.56)	[30, 31]
Recurrence	0–5	0.66	[28]
	6–10	0.65	
	11–15	0.61	
Metastasis	0–5	0.44	[28]
	6–10	0.43	
	11–15	0.41	

The costs of different WBRT techniques from payer perspective were based on local hospital Medicare charges, including costs for physician consultant, dosimetry, treatment fractions, physics quality assurance etc., and are shown in Table 2, and the costs of treating 3 radiogenic late side effects were extracted from literature and are also included in Table 2. Only direct medical costs are considered in this study. The advanced techniques contain radiation intensity modulation, which requires a specific charge for the equipment and extra workload for quality assurance performed by medical physicists. This is the major reason that advanced RT techniques are more expensive than SOC. Costs and utilities were discounted at an annual rate of 3% [32].

**Table 2:** Treatment costs.

Treatment	Cost (range)	Reference
WBRT (SOC/FIF)	$12,140	Based on Medicare charge
WBRT (Hybrid IMRT)	$15,293	Based on Medicare charge
WBRT (VMAT/ IMRT)	$17,438	Based on Medicare charge
Cardiac toxicity	$11,570±3405	[29, 33]
CL breast cancer	$14,494±1199	[34]
Lung cancer	$20,577±2740	[35–37]
local recurrence	$20,879	[38]
Metastasis	$13,627	[38]

Since the clinical data of tumor control or radiogenic late effects after advanced WBRT techniques were largely incomplete, we assumed probabilities of local recurrence and distant metastasis for advanced WBRT to be the same as those after SOC WBRT, and calculated probabilities of radiogenic late effects for those techniques using well-defined risk models including lifetime attributable risk (LAR) for second cancers [39] and risk for coronary events (RCE) [40, 41] as shown in Table 3. We assumed that all lung and cardiac events start from year 11 after radiotherapy [40, 42], while contralateral breast events start from year 6 after radiotherapy [43].

**Table 3:** Calculated annual probabilities of developing radiogenic side effects for the 65-year-old cohort from a previous study [44].

Side effect	Probability (%)	Range (%)
SOC cardiac toxicity	1.24	0.80–2.03
SOC CL breast cancer	0.38	0.13–1.39
SOC lung cancer	0.22	0.18–0.28
FIF cardiac toxicity	1.15	0.80–1.97
FIF CL breast cancer	0.38	0.18–0.71
FIF lung cancer	0.22	0.18–0.31
Hybrid IMRT cardiac toxicity	1.16	0.77–1.88
Hybrid IMRT CL breast cancer	0.35	0.12–1.33
Hybrid IMRT lung cancer	0.20	0.16–0.27
IMRT cardiac toxicity	1.14	0.74–1.78
IMRT CL breast cancer	0.32	0.71–0.71
IMRT lung cancer	0.20	0.14–0.27
STD-VMAT cardiac toxicity	1.16	0.69–1.77
STD-VMAT CL breast cancer	0.27	0.17–0.54
STD-VMAT lung cancer	0.20	0.18–0.29
NC-VMAT cardiac toxicity	1.05	0.65–1.51
NC-VMAT CL breast cancer	0.27	0.17–0.78
NC-VMAT lung cancer	0.18	0.13–0.25
MA-VMAT cardiac toxicity	1.03	0.65–1.52
MA-VMAT CL breast cancer	0.26	0.15–0.39
MA-VMAT lung cancer	0.17	0.10–0.24

The model validity was assessed by comparing 15-year survival results with predicted results from CancerMath, which is the latest web-based prognostic tool that includes conventional radiotherapy as part of treatment for breast cancer patients [45, 46].

### Sensitivity analyses

A series of one-way sensitivity analyses were performed over a wide range of assumptions for probabilities, utilities, and treatment costs of radiogenic side effects for six advanced WBRT techniques versus SOC.

Probability sensitivity analyses (PSA) were also performed to assess the uncertainty and robustness of the model by assigning specific distributions for model parameters, where the probabilities, utilities, and costs of radiogenic side effects were varied simultaneously across their distributions using Monte Carlo simulation. Recommended by Briggs *et al*. [47], we used beta distribution for transition probabilities and utilities estimates, and used gamma distributions for cost parameters. The cost-effectiveness acceptability was calculated based on the result of 100,000 simulations for each WBRT technique at different WTP thresholds.

## Results

The external validation of our model was assessed for 65-year-old women with early-stage breast cancer. Our model predicted a 15-year overall survival rate of 53.3% and breast cancer mortality rate of 21.7%, and CancerMath calculated an overall survival rate of 55.0% and breast cancer mortality rate of 19.5%. These comparisons suggest that our model’s predictions are similar to real clinical outcomes.

Table 4 shows baseline ICERs for all six WBRT techniques compared with SOC. Among six techniques that been analyzed, FIF shows the lowest ICER of $1,511/QALY while IMRT shows highest ICER of $121,087/QALY.

**Table 4:** Cost, quality-adjusted life-years (QALY), and incremental cost-effectiveness ratio (ICER) for advanced PMRT techniques compared with SOC.

	SOC	FIF	Hybrid	IMRT	STD-VMAT	NC-VMAT	MA-VMAT
**Costs ($)**	16,239	16,242	18,742	19,145	20,510	20,540	20557
**QALY**	6.408	6.410	6.437	6.432	6.451	6.452	6.455
**ICER ($/QALY)**	-	1,511	86,316	121,087	99,315	97,759	91,872

The one-way analyses (S1 Fig) show that the cost-effectiveness of all six advanced WBRT techniques is most sensitive to the probability of developing contralateral breast cancer. S2 Fig shows cost-effectiveness acceptability curves for various advanced WBRT techniques at different willingness to pay thresholds. The probabilities of being more cost-effective than SOC for six WBRT techniques are shown in Table 5. At a WTP threshold of $50,000/QALY, except FIF which has a 58.9% probability of being more cost- effective, none of the other five WBRT techniques has an over 2.0% probability of being more cost-effective. At a WTP threshold of $100,000/QALY, FIF, hybrid IMRT and MA-VMAT are more likely to be cost-effective than SOC with a probability of 59.2%, 72.3% and 72.6%, respectively.

**Table 5:** Probability of being more cost-effective than SOC for advanced techniques.

WTP ($/QALY)	FIF	Hybrid	IMRT	Std-VMAT	NC-VMAT	MA-VMAT
50,000	58.9%	2.0%	0.0%	0.5%	0.5%	0.0%
100,000	59.2%	72.3%	12.9%	44.9%	56.6%	72.6%

## Discussion

This study presents the most comprehensive cost-effectiveness analysis of seven WBRT techniques. Not only tumor control but also radiogenic side effects were included in the study. IMRT shows the highest baseline ICER which may due to its higher initial treatment cost and limited improvement of normal tissue sparing. FIF has the lowest baseline ICER which is mainly due to its low initial treatment cost and relatively lower probability of inducing cardiac toxicity compared with SOC. As shown in the PSAs, FIF, hybrid IMRT and MA-VMAT are more likely to be more cost- effective than SOC and other WBRT techniques at a WTP of $100,000/QALY, while SOC WBRT appeared to be more cost-effective than advanced WBRT techniques except for FIF at WTP of $50,000/QALY. Given the prevalence of breast cancer and continued growth of health care costs, results from this analysis will have a positive impact. Prior study has shown that up to 30% of the medical care spending in the US is unnecessary or inappropriate [48]. Moreover, study on Medicare patients found that higher spending was associated with more care but not better health outcomes [49]. The results from our analysis may benefit health care professionals and help choose the most cost-effective health intervention for breast cancer patients.

Radiotherapy is a crucial component of breast cancer treatment, and several studies have compared cost-effectiveness between different WBRT and accelerated partial breast irradiation (APBI) techniques. Shah *et al*. [18] reported that external beam APBI costs less and is more effective than hypofractionated WBRT after 90 days of the initial treatment. Another study by their group [50] compared multiple APBI techniques with WBRT delivered with 3D-conformal therapy and IMRT, and showed that external beam APBI is more cost-effective than WBRT using 3D-conformal therapy, and all APBI techniques are more cost-effective than WBRT using IMRT. Sher *et al*. [17] compared the cost-effectiveness between WBRT, external beam and MammoSite (MS) APBI, and also concluded that external beam APBI is the most cost- effective strategy for early-stage breast cancer patients. However, not all lumpectomy patients are eligible for APBI, and the cost-effectiveness analysis of different advanced WBRT techniques is largely lacking. Sen *et al*. [51] compared no radiotherapy, conventional external beam WBRT and IMRT WBRT for women older than 70. They only considered tumor control, and they reported that IMRT would have to be substantially more effective in improving quality of life than conventional external beam therapy to be cost-effective. Because they did not include short-term or long-term side effects in their study, they used the increase of utility of baseline state to be a vague representation of improvement in quality of life. Our study shows that the risk of developing contralateral breast cancer may significantly affect the cost-effectiveness for advanced WBRT techniques, and contralateral breast cancer is directly related to quality of life for WBRT patients. Our study is therefore consistent with Sen *et al*. [51] and clearly suggests reducing irradiation of contralateral breast could be a key factor to make advanced WBRT technique more cost-effective. For SOC WBRT, considerable volumes of heart and ipsilateral lung are likely to receive high doses which may lead to radiation-related toxicities such as secondary lung cancer [52] and heart disease [40, 53]. The radiogenic side effects will not only affect patients’ quality of life, but also add further economic burden for WBRT patients. Advanced radiotherapy techniques could avoid high dose expose to surrounding and underlying healthy tissues and improve the quality of life for the patients.

A previous study from our group evaluated cost-effectiveness of current SOC post-mastectomy radiotherapy (PMRT) and seven advanced PMRT techniques over 15 years [54]. It used a similar Markov model and showed that the model outcomes are most sensitive to the probability of developing cardiac toxicity for PMRT patients. As shown in the one-way analysis in this study, the cost-effectiveness of all WBRT techniques is most sensitive to the risk of developing contralateral breast cancer. The possible reason for this discrepancy is treatment target is much closer to heart for PMRT patients than for WBRT patients, which significantly increases the risks of heart toxicity and makes it a contributing factor to the cost-effectiveness of PMRT techniques. On the other hand, the risk of developing contralateral breast cancer for WBRT patients is much higher than for PMRT patients, which is mainly due to the relatively large field size used in WBRT to cover the whole ipsilateral breast and increased irradiation of the contralateral breast. Additionally, treating contralateral breast cancer will cost almost $3,000 more than treating heart toxicity (Table 2). Therefore, providing better sparing of contralateral breast is essential for advanced WBRT technique to be cost-effective.

Proton therapy was not included in this study. Although proton therapy has been shown to be a promising WBRT technique to reduce heart and lung dose [55, 56], it did not gain popularity due to its limited availability¸ multiple uncertainties [57, 58], possible skin toxicity [59, 60] and significantly higher cost. Another effective method to limit radiation dose to heart and lung is deep inspiration breath-hold (DIBH), which is particularly useful for treating patients with left-sided breast cancer [61, 62]. Macrie *et al*. [63] reported a DIBH program that is inexpensive to implement and has minimum influence on patient throughput. Chatterjee *et al*. [64] concluded that although DIBH requires significant resource commitments regarding person-hours, it is still more cost-effective due to the reductions in cardiac mortality. Comparing cost-effectiveness of various WBRT techniques for patients with DIBH is still lacking and will be investigated by our group in the near future. Hypofractionated WBRT is effective for selected early-stage breast cancer patients. It is more convenient for the patients and caregivers and can significantly cut treatment costs. However, hypofractionation is not the current SOC in our clinic or many other clinics in the US. Considering the cost calculations will be completely different, the outcome data for patients treated with hypofractionation are scarce, and existing dose-risk models are based on standard fractionated radiotherapy, we did not include those patients in this study. The cost-effectiveness evaluation of various techniques for hypofractionated WBRT patients will be investigated by our group in the future when more outcome data come out.

Our study has some limitations. First, we used well-defined dose-risk models to calculate probabilities of developing radiogenic late effects for advanced WBRT techniques because there is a lack of clinical outcome data. However, as shown in another study from our group [44], the calculated risks of cardiac toxicity and second cancers after SOC WBRT are in good agreement with the clinical outcomes, so we expect our calculated risks values for advanced WBRT techniques are also reasonable. Long-term clinical trial outcomes will be needed to validate the calculated outcomes for advanced WBRT techniques. Second, it’s difficult to assess the total costs of care for primary cancer or treatment-related side effects from the available literatures. As Campbell and Ramsey [65] point out, many studies ignored or truncated the duration of the continuing care period, or only focused on certain subpopulations, and many of them are quite dated and do not reflect changes in patterns of care. It is possible that the outcome and cost values used in our study contain uncertainties, but the comprehensive uncertainty analyses took these into account by varying each possible uncertain value over plausible ranges and assessing their impact on the final cost-effectiveness.

## Conclusions

We evaluated cost-effectiveness of seven WBRT techniques. Based on calculated ICER values and comprehensive uncertainty analyses, FIF appears to be the most cost-effective approach for WBRT patients at a WTP threshold of $50,000/QALY, and hybrid IMRT and MA-VMAT might be the most cost-effective options at a WTP threshold of $100,000/QALY. Providing better sparing of contralateral breast is essential for advanced WBRT techniques to be cost effective.

## Supporting information

S1 Fig Tornado diagram of one-way analyses that compare SOC with (a) FIF, (b) Hybrid, (c) IMRT, (d) STD-VMAT, (e) NC-VMAT and (f) MA-VMAT. One-way sensitivity analysis examines the impact of variables on the outcomes (e.g., ICER) by changing a specific value over its uncertainty range while keeping all other variables constant at their baseline value. The dashed line represents the baseline ICER, and the width of the bars represents the change of ICER based on the uncertainty range of each variable. The wider the bar is, the more significant impact the variable has on the ICER value. P_: probability of developing certain radiogenic side effect using certain WBRT technique. Utility_: utility value for certain radiogenic side effect. Cost_: cost of treating certain radiogenic side effect.
(DOCX)

S2 Fig Cost-effectiveness acceptability curves that compare the cost-effectiveness of SOC and (a) FIF, (b) Hybrid, (c) IMRT, (d) STD-VMAT, (e) NC-VMAT and (f) MA-VMAT at different willingness to pay (WTP) thresholds. The data points indicate the percentage of iterations out of 100,000 iterations that were cost-effective at a given WTP threshold during probabilistic sensitivity analysis. The two dashed lines shown in the figures highlight the WTP threshold at 50,000 $/QALY and 100,000 $/QALY, respectively.
(DOCX)
