# Supplementary material for: Cost-effectiveness analysis of radiotherapy techniques for whole breast irradiation
Source: PLoS One. 2021 Mar 8;16(3):e0248220. doi: 10.1371/journal.pone.0248220 (PMC7939353; doi:10.1371/journal.pone.0248220)
Supplement: S1 Fig — One-way sensitivity analysis examines the impact of variables on the outcomes (e.g., ICER) by changing a specific value over its uncertainty range while keeping all other variables constant at their baseline value. The dashed line represents the baseline ICER, and the width of the bars represents the change of ICER based on the uncertainty range of each variable. The wider the bar is, the more significant impact the variable has on the ICER value. P_: probability of developing certain radiogenic side effect using certain WBRT technique. Utility_: utility value for certain radiogenic side effect. Cost_: cost of treating certain radiogenic side effect. (DOCX) [file pone.0248220.s001.docx]

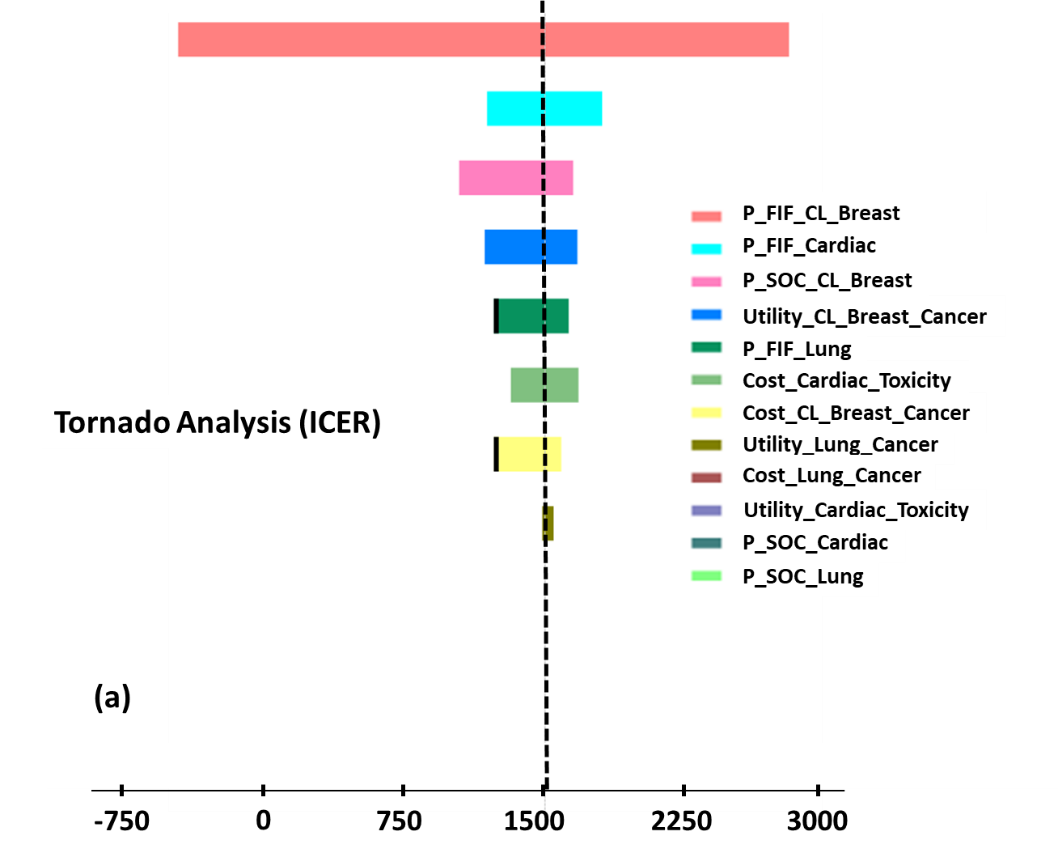


**ICER ($/QALY)**


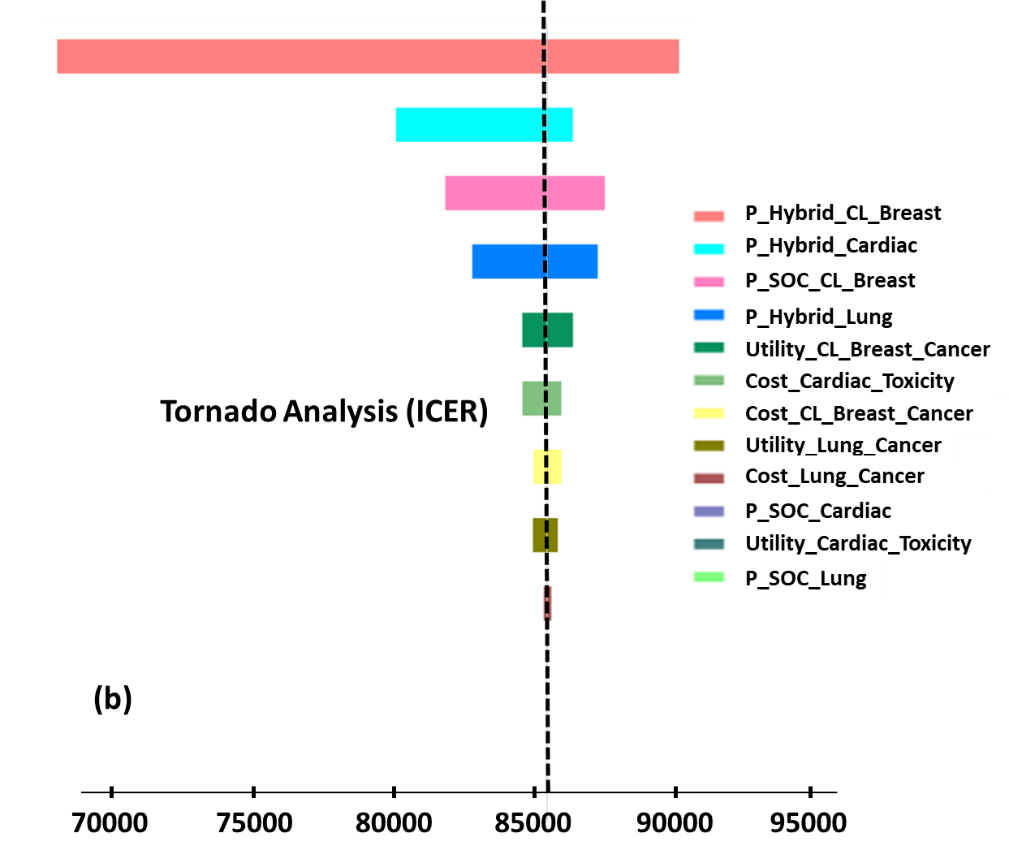


**ICER ($/QALY)**


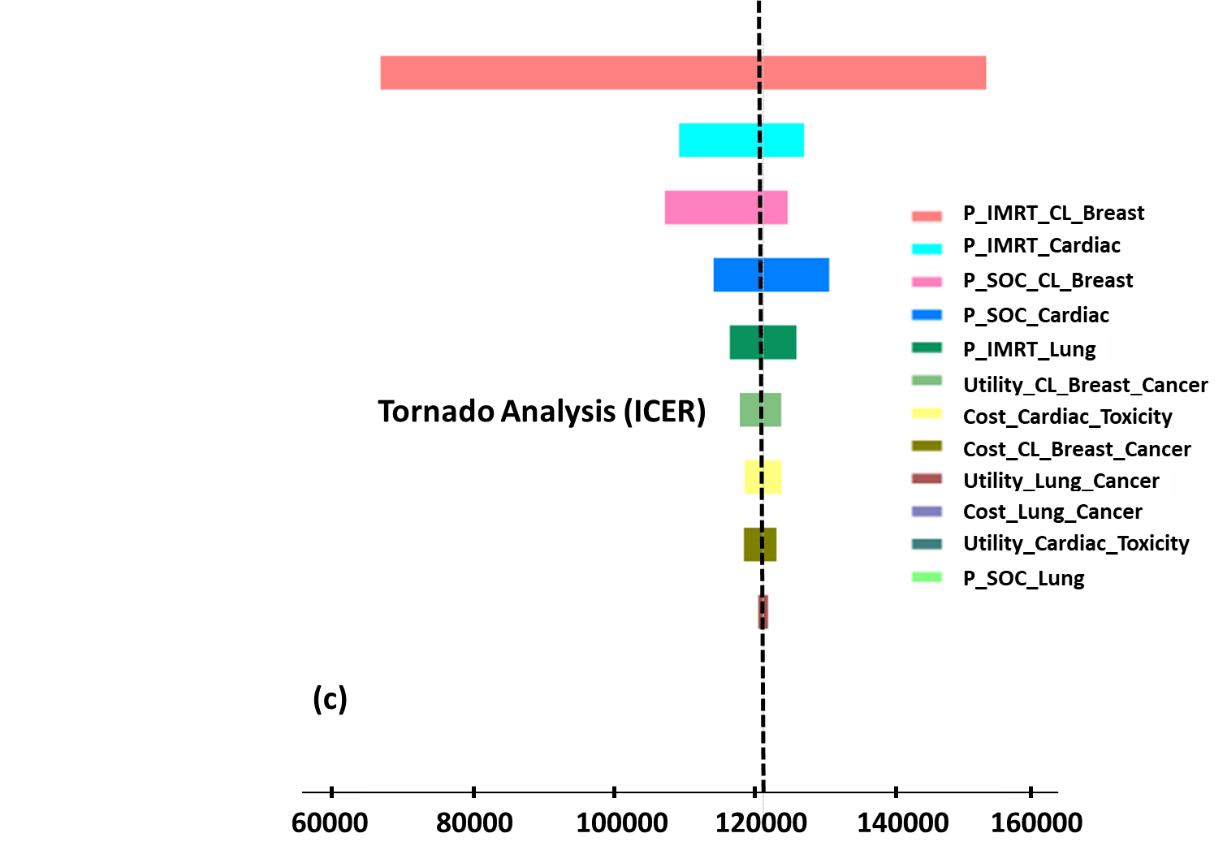


**ICER ($/QALY)**


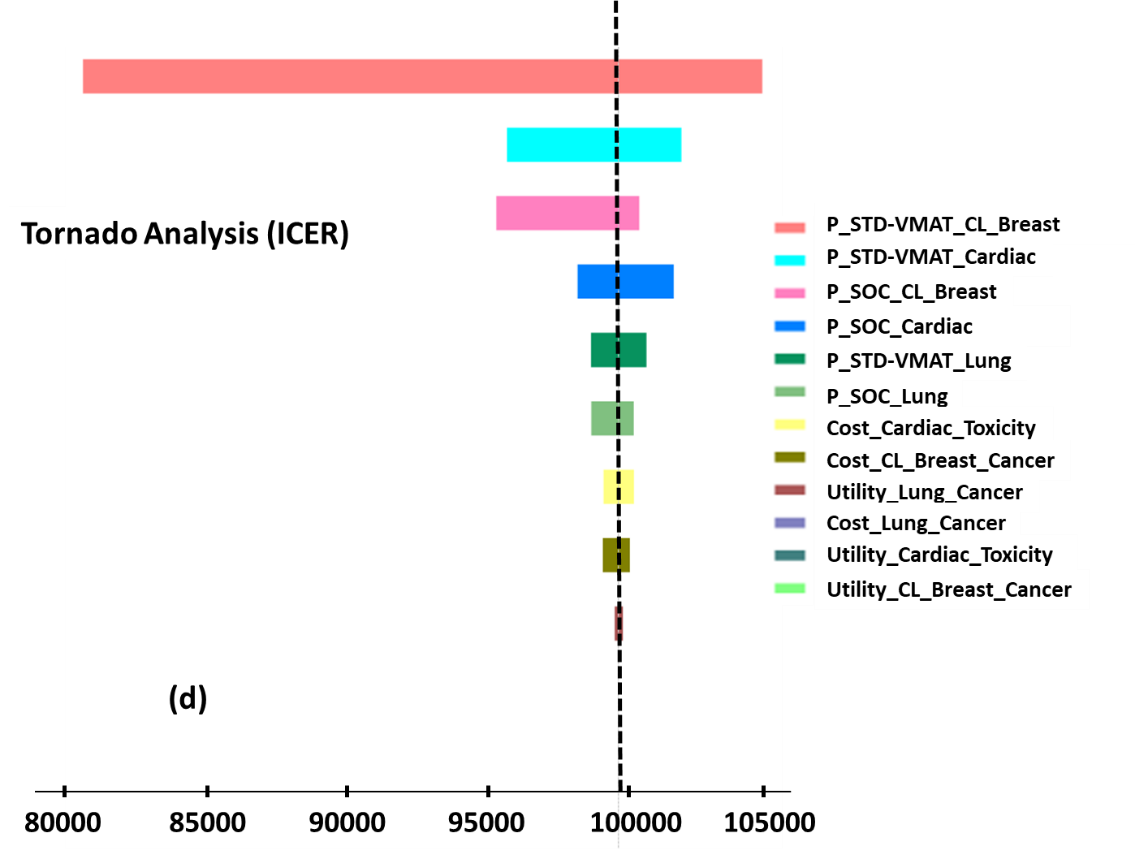


**ICER ($/QALY)**


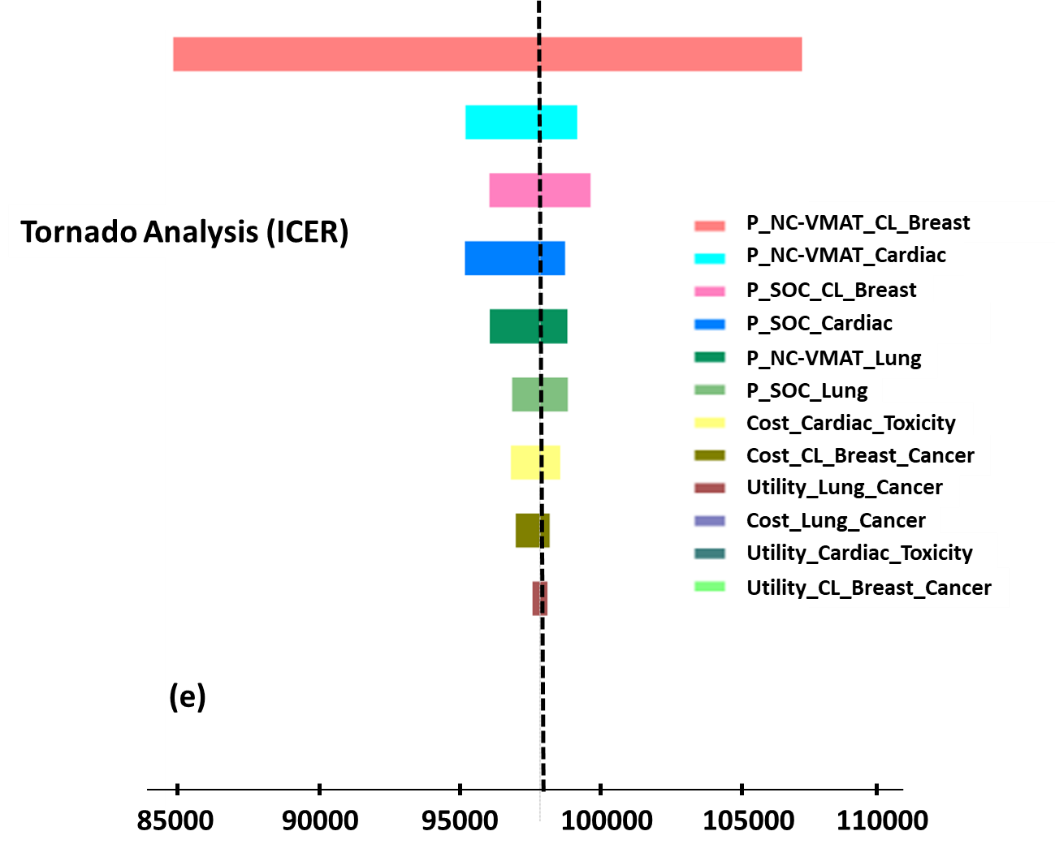


**ICER ($/QALY)**


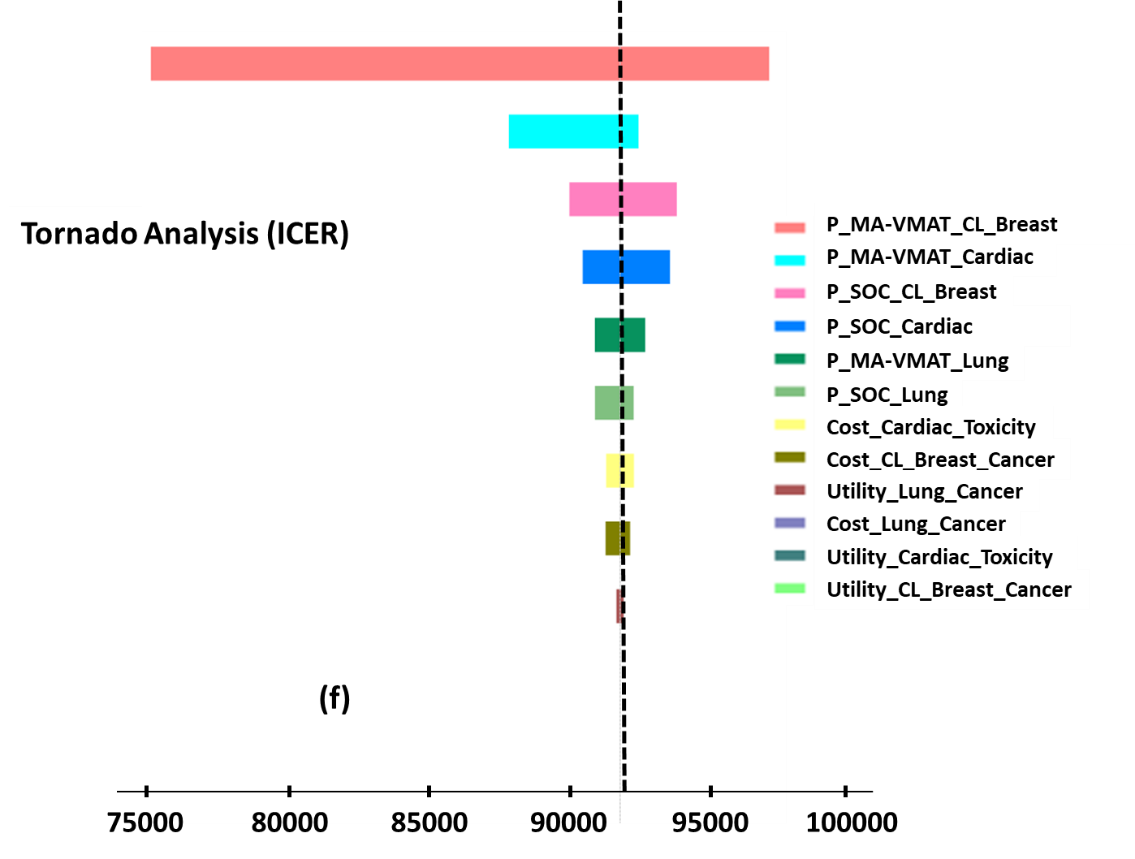


**ICER ($/QALY)**

**S1 Fig.** Tornado diagram of one-way analyses that compare SOC with (a) FIF, (b) Hybrid, (c) IMRT, (d) STD-VMAT, (e) NC-VMAT and (f) MA-VMAT. One-way sensitivity analysis examines the impact of variables on the outcomes (e.g., ICER) by changing a specific value over its uncertainty range while keeping all other variables constant at their baseline value. The dashed line represents the baseline ICER, and the width of the bars represents the change of ICER based on the uncertainty range of each variable. The wider the bar is, the more significant impact the variable has on the ICER value. P_: probability of developing certain radiogenic side effect using certain WBRT technique. Utility_: utility value for certain radiogenic side effect. Cost_: cost of treating certain radiogenic side effect.
