# Supplementary material for: Cost-effectiveness analysis of radiotherapy techniques for whole breast irradiation
Source: PLoS One. 2021 Mar 8;16(3):e0248220. doi: 10.1371/journal.pone.0248220 (PMC7939353; doi:10.1371/journal.pone.0248220)
Supplement: S2 Fig — The data points indicate the percentage of iterations out of 100,000 iterations that were cost-effective at a given WTP threshold during probabilistic sensitivity analysis. The two dashed lines shown in the figures highlight the WTP threshold at 50,000 $/QALY and 100,000 $/QALY, respectively. (DOCX) [file pone.0248220.s002.docx]

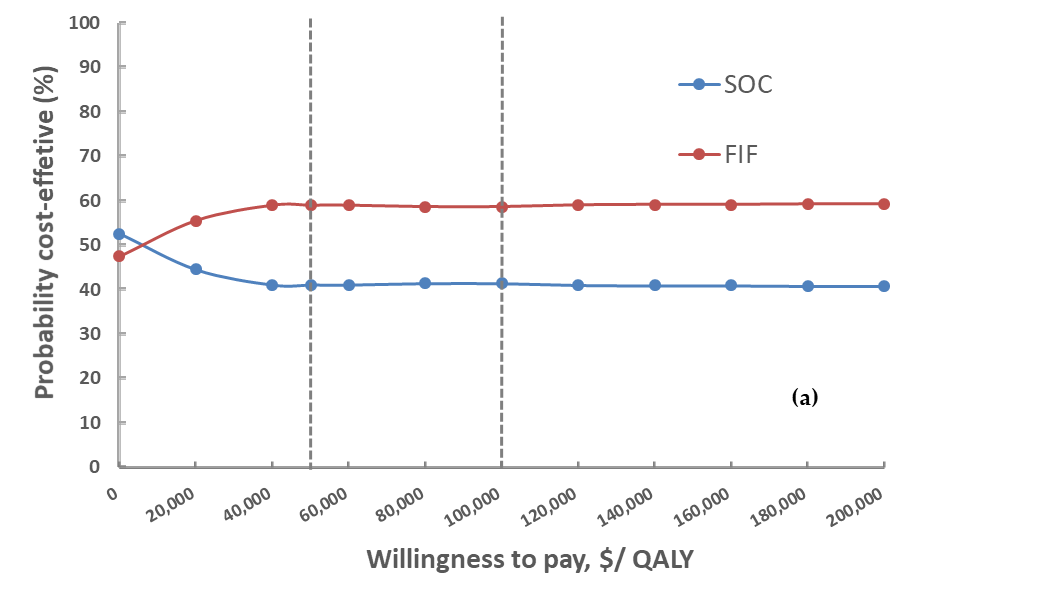


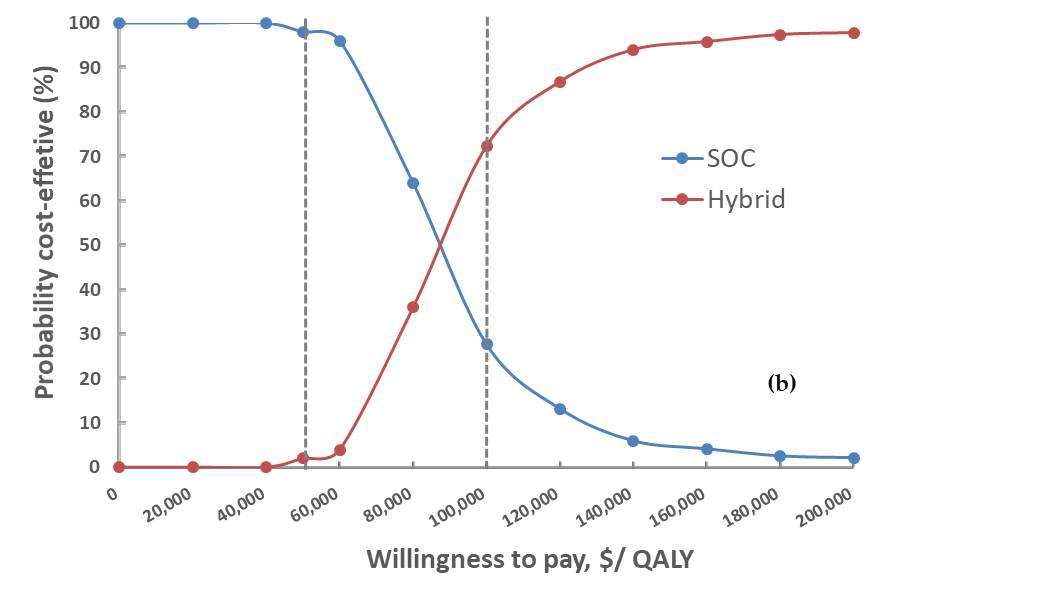


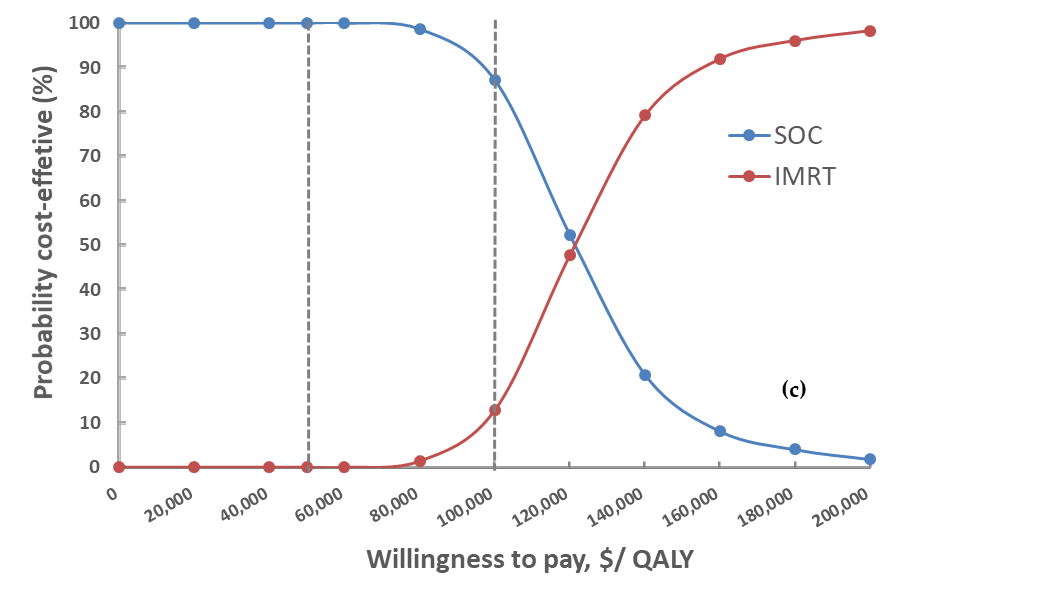

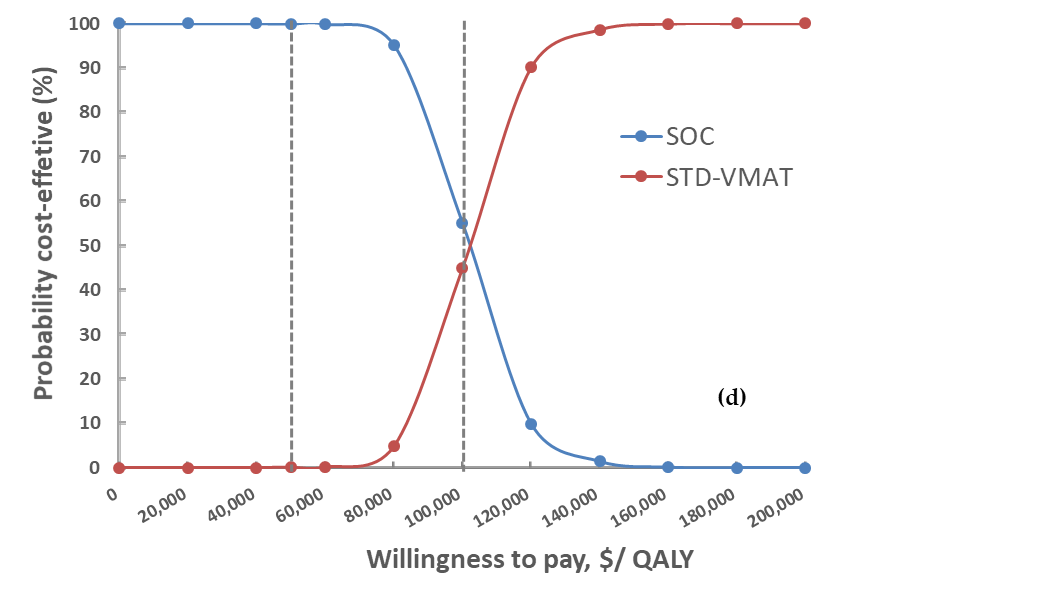
 **
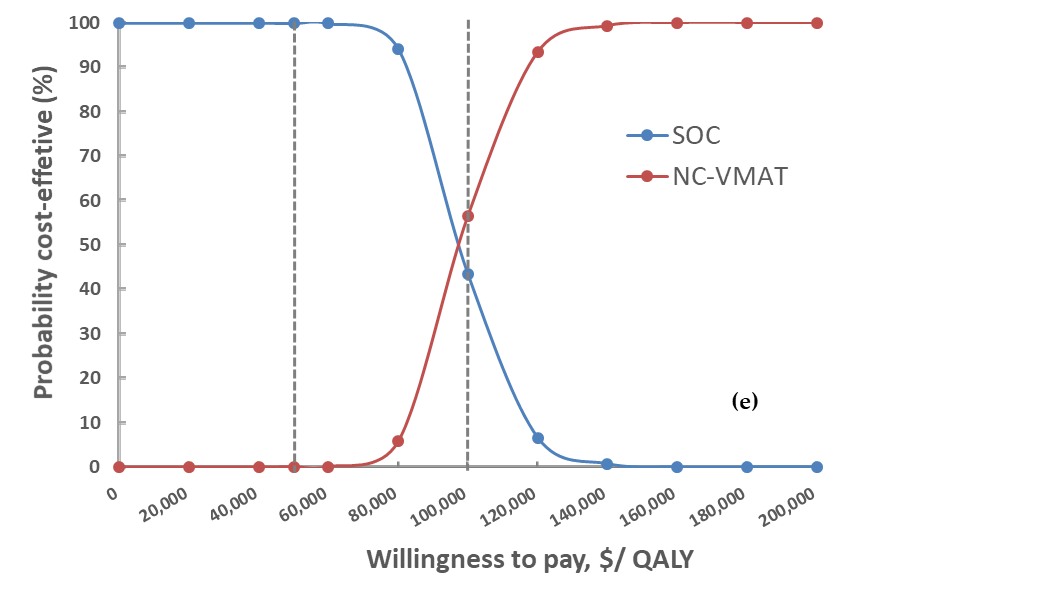
**

**
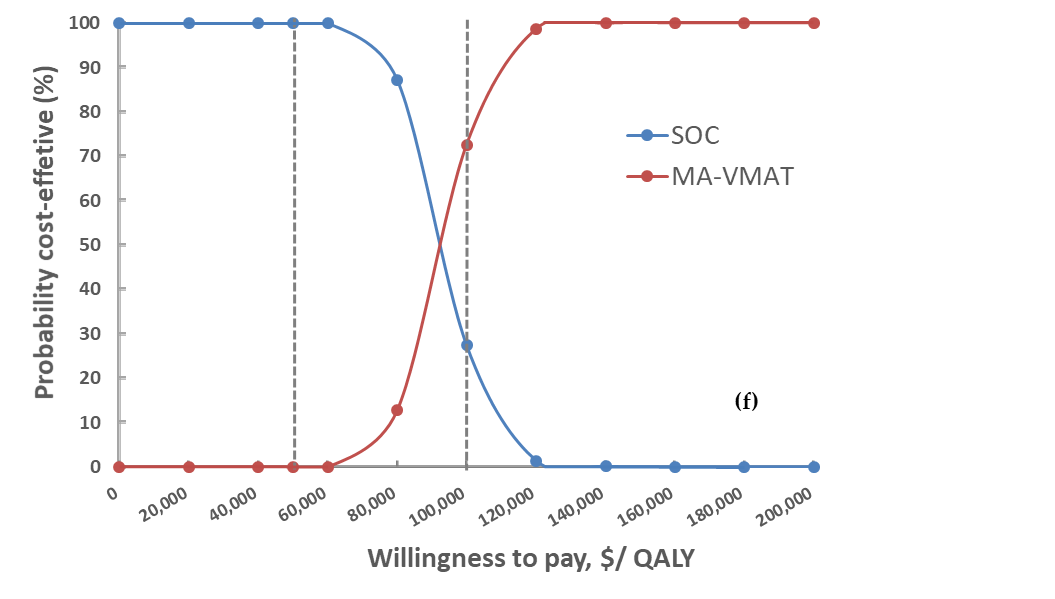
**

**S2 Fig.** Cost-effectiveness acceptability curves that compare the cost-effectiveness of SOC and (a) FIF, (b) Hybrid, (c) IMRT, (d) STD-VMAT, (e) NC-VMAT and (f) MA-VMAT at different willingness to pay (WTP) thresholds. The data points indicate the percentage of iterations out of 100,000 iterations that were cost-effective at a given WTP threshold during probabilistic sensitivity analysis. The two dashed lines shown in the figures highlight the WTP threshold at 50,000 $/QALY and 100,000 $/QALY, respectively.
